# Pregelatinized Starch-Based Edible Films as Effective Carriers for *Bacillus coagulans*: Influence of Starch Type on Film Properties and Probiotic Viability

**DOI:** 10.3390/foods14142424

**Published:** 2025-07-09

**Authors:** Laily Dwi Rahma, Atcharawan Srisa, Phanwipa Wongphan, Massalin Nakphaichit, Shyam S. Sablani, Nathdanai Harnkarnsujarit

**Affiliations:** 1 Department of Packaging and Materials Technology, Faculty of Agro-Industry, Kasetsart University, 50 Ngam Wong Wan Rd., Latyao, Chatuchak, Bangkok 10900, Thailand; lailydwi.r@ku.th (L.D.R.); atcharawan.sri@ku.th (A.S.); phanwipa.w@ku.th (P.W.); 2 Department of Biotechnology, Faculty of Agro-Industry, Kasetsart University, 50 Ngam Wong Wan Rd., Latyao, Chatuchak, Bangkok 10900, Thailand; fagimln@ku.ac.th; 3 Biological Systems Engineering Department, Washington State University, Pullman, WA 99164-6120, USA; ssablani@wsu.edu

**Keywords:** edible film, probiotic, carrier, pregelatinized starch, hydroxypropyl starch, *Bacillus coagulans*

## Abstract

Incorporating probiotics into edible films offers an effective strategy for delivering viable microorganisms to the body. This study aimed to develop edible films based on three types of pregelatinized cassava starch—pregelatinized native starch (PNS), hydroxypropyl distarch phosphate (HDP), and hydroxypropyl starch (HS)—as carriers for *Bacillus coagulans* (BC). The interactions between probiotic powder and the polymer matrix, as well as the viability of *B. coagulans* during film drying and subsequent storage, were evaluated to assess the effectiveness of the films as protective delivery systems at room temperature (25 °C). The addition of BC altered the amorphous-to-ordered structure of the starch matrices. Surface morphology analysis showed BC aggregates on PNS films, whereas HDP and HS films retained smooth surfaces. Incorporation of BC increased the tensile strength and Young’s modulus of PNS films but reduced their elongation at break. Additionally, BC decreased both the light transmittance and water contact angle in PNS films, while 1% BC increased the contact angle in HDP and HS films. BC had no significant effect on the solubility of PNS films but enhanced the solubility of HDP and HS films. Notably, *B. coagulans* maintained viability around 8 log CFU/g after 90 days of storage at room temperature, supporting the potential of pregelatinized starch-based films as effective probiotic carriers.

## 1. Introduction

Starch is a low-cost, renewable biopolymer capable of forming continuous film matrices. Cassava is one of various sources for producing cost-effective starch. However, it exhibits poor flow properties, high cohesiveness, and low resistance to shear, high temperatures, and acidic conditions which limits its use in various industrial applications [1,2]. Pregelatinized cassava starch undergoes irreversible granule swelling and loss of crystallinity, resulting in improved flowability, compaction, water absorption, cold-water solubility, and swelling power, which help overcome the limitations of the native starch [1,2,3]. Pregelatinized starch refers to starch that has been modified through physical, chemical, and enzymatic changes. During the pregelatinization process, the amylose and amylopectin in starch granules are disrupted, decreasing crystallinity and enhancing water absorption capacity, which contributes to excellent cold-water swelling behavior. Therefore, pregelatinized starch is particularly advantageous for producing edible films at low temperatures [4,5,6]. Edible films offer notable advantages as carriers for bioactive compounds, which can enhance packaging functionality by enhancing health benefits or reduced health risks. These thin-layer films have been developed to deliver specific nutrients, pharmaceuticals, and food ingredients—one of the most promising being probiotics [3,7].

Probiotics are live microorganisms that, when consumed in adequate amounts, provide health benefits to the host [8,9]. They are non-pathogenic and generally recognized as safe (GRAS), posing no risk to food safety. Probiotic functions such as antimicrobial and antiviral activity, enhancement of the intestinal barrier and immune modulation are particularly valuable [10]. Probiotics are commonly consumed through fermented and non-fermented foods or dietary supplements in powder, tablet, or capsule form [11]. A novel strategy involves incorporating probiotics into plasticized hydrocolloids-based edible films, which offers a promising method for enhancing probiotic stability and delivery. As a result, research into probiotic delivery systems and novel applications has grown rapidly [12,13].

Incorporating probiotics into edible films is an emerging technique for improving their viability and survivability during the food production process. This approach helps addresses challenges related to the survival of probiotics, which are sensitive to chemical and physical factors [7]. Karimi, et al. developed edible films based on whey protein isolate/polydextrose and cellulose nanofiber (CNF) containing *Lactobacillus plantarum* [14]. The result showed that incorporating *L. plantarum* into edible film retained its viability after the drying process, with viability increased by the incorporation of polydextrose and CNF. Similarly, Orozco-Parra, et al. demonstrated the protective effect of starch and inulin on *Lactobacillus casei* during film drying process [9]. Oliveira-Alcântara, et al. also showed that bacterial cellulose/cashew gum/fructooligosaccharides films improved the viability of *Bacillus coagulans* during drying process and 45 days of storage at 4 °C and 20 °C [15]. Despite these advancements, studies specifically focusing on the incorporation of *Bacillus coagulans* into edible films remain limited [8,15,16,17]. However, no previous studies have explored the use of pregelatinized cassava starch as a carrier for *Bacillus coagulans* in edible films, despite its advantages such as room-temperature processability and suitability for heat-sensitive probiotic applications.

In this study, three types of pregelatinized cassava starch, namely pregelatinized native cassava starch (PNS), pregelatinized modified cassava starch INS1442 (HDP), and pregelatinized modified cassava starch INS1440 (HS) were used as probiotic carrier matrices. *Bacillus coagulans* was incorporated into starch films via solution casting at three different concentrations (0, 1 and 1.5% *w*/*w*). The effects of incorporating *B. coagulans* were evaluated using scanning electron microscopy (SEM), Fourier-transform infrared spectroscopy (FTIR), and mechanical property testing to clarify the interaction between the probiotics and the polymer matrix. In addition, the survivability of *B. coagulans* during storage was assessed to determine the effectiveness of the edible films as probiotic carriers during storage at room temperature. This study aims to provide insight into optimizing starch-based films for applications in food and feed products.

## 2. Materials and Methods

### 2.1. Materials

Pregelatinized native cassava starch (PNS), pregelatinized hydroxypropyl distarch phosphate (HDP), and pregelatinized hydroxypropyl starch (HS) were purchased from Siam Modified Starch Corporation Co., Ltd. (Pathum Thani, Thailand). *Bacillus coagulans* BACO-17 (BC) is a spore-forming, Gram-positive, rod-shaped probiotic strain was purchased from Syngen Biotech Co., Ltd. (Tainan, Taiwan). The bacterial powder had a declared viability of 5 × 10^9^ spores per gram dry powder. Lactobacillus DeMan, Rogosa, Sharpe (MRS) agar (M641) was purchased from HiMedia (Mumbai, India). Glycerol and potassium phosphate (KH_2_PO_4_) were purchased from Ajax Finechem Pty., Ltd. (Sydney, Australia). Sodium hydroxide (NaOH, KA482) was obtained from KemAus (Cherrybrook, Australia).

### 2.2. Film Preparation

Three different pregelatinized starches, PNS, HDP, and HS at 5% (*w*/*w*) were dissolved in 100 g of distilled water at room temperature (25 °C) and left for 24 h. Glycerol at 35% of starch weight (*w*/*w*), and BC at concentrations of 0, 1, and 1.5% (*w*/*w*) were then added to the solution and mixed using a magnetic stirrer (C-MAG HS 7, IKA Works GmbH & Co., KG, Staufen, Germany) for 1 h. A 20 g and 40 g mixture were poured into 9 cm and 14 cm diameter polystyrene petri dishes, respectively. Films from 9-cm petri dishes were used for most tests (e.g., morphology, thickness, solubility, probiotic viability), while 14-cm dishes were used for oxygen permeability and tensile tests requiring larger sample sizes, following ASTM standards. Films were then dried in a hot air oven (FD 53, Binder GmbH, Tuttlingen, Germany) for 24 h at 40 °C. The dried film was detached from the dish and stored in a controlled humidity chamber at 50% RH, 25 °C at least 48 h before measurement.

### 2.3. Fourier-Transform Infrared Spectroscopy (FTIR)

The interaction between BC powder and film materials was analyzed using FTIR. Infrared (IR) spectra of the films were obtained using attenuated total reflectance Fourier transform infrared spectroscopy (ATR, FTIR) mode (Nicolet iS10 FTIR Spectrometer, Thermo Fisher Scientific, Waltham, MA, USA). The IR absorbance spectra were recorded in the range of 4000–650 cm^−1^ with a resolution of 4 cm^−1^ and averaged over 64 scans and standardized with air spectrum [18]. Measurements were performed in triplicate for each film formulation.

### 2.4. Microstructures

Scanning electron microscopy (SEM) (SU3500 SEM, Hitachi Co., Ltd., Matsuda, Japan) was used to characterize the cross-sectional and surface microstructures of the films. Samples were prepared by fracturing the films in liquid nitrogen to obtain clean cross-sections and surface exposures. The specimens were mounted on copper stubs and sputter-coated with gold. SEM images of both the surface and cross-section were acquired at 1500× magnifications of using an accelerating voltage of 5 kV (method modified from [3]).

### 2.5. Light Transmission

Light transmission tests were conducted to evaluate the effect of BC powder addiction on the optical properties of the films. Measurements were performed using a UV-Vis spectrophotometer (Evolution 300, Thermo Fisher Scientific, Waltham, MA, USA). Film samples (3 × 4 cm) were placed between metal plates with a slit, and the light transmission was recorded across the 200–800 nm wavelength range at a scan speed of 240 nm/min. Each treatment was tested in triplicate [3].

### 2.6. Mechanical Properties

Mechanical properties of the films were determined according to ASTM D882-10 [19] using an Instron universal testing machine (Model 5965, Instron, Canton, MA, USA). Samples were cut into 2.5 × 10 cm strips, and thickness was measured at five points per strip. At least five specimens per treatment were tested (*n* = 5). The grip separation distance was set to 50 mm with a crosshead speed of 500 mm/min. Tensile strength, elongation at break, and Young’s modulus were measured [20].

### 2.7. Surface Hydrophobicity

Surface hydrophobicity was measured using contact angle (CA) measurement device (OCA 15 EC, Data physics Instruments GmbH, Filderstadt, Germany). A 3 μL droplet of distilled water was deposited onto the film surface (1 × 10 cm) using a micro syringe, and the contact angle was recorded immediately within 1 s using the SCA 20 software [3]. All measurements were performed at room temperature (25 ± 1 °C) with ten replicates per treatment.

### 2.8. Water Vapor Permeability

The water vapor permeability of the films was determined according to the standard cup method (ASTM E96) [21] with five replications. Films were cut into 7 cm circular discs and sealed over aluminum cups containing dried silica gel, using O-rings and molten paraffin. The sample was stored in a humidity chamber at 50% RH, 25 °C and weighed until the weight was constant. The water vapor transmission rate (WVTR) (g/m^2^·day) was calculated from the linear slope of weight gain over time and water vapor permeability (WVP) was calculated using Equation (1):

WVP (g·mm/m^2^·day·kPa) = (WVTR × L)/ΔP (1)

where L is the film thickness (mm) and ΔP is the partial pressure difference (kPa) between the two sides of the film [3].

### 2.9. Water Solubility

Water solubility of the films was measured according to the method of Wongphan and Harnkarnsujarit [3] with some modification. Film samples (2 × 2 cm) were first dried for 24 h at 70 °C to standardize initial moisture content across all treatments. The dried films were weighed (W_1_) and then immersed in 30 mL of distilled water at room temperature for 24 h. After immersion, the remaining undissolved films were collected, drained, and dried at 70 °C for 24 h. The final dry weight (W_2_) was recorded carefully without absorbing any moisture. Water solubility was calculated with Equation (2):

(2) $$\mathrm{Water}\;\mathrm{solubility}\;(\%)=\frac{W_{1}-W_{2}}{W_{1}}\;\times \;100$$

### 2.10. Viability of Probiotic Before and After Drying Process

The viability of probiotics in film solution and dried films was measured using the pour plate method according to Karimi, Alizadeh, Almasi and Hanifian [14] with some modification. For the film solution, 1 mL was diluted in 9 mL sterile Butterfield’s phosphate buffer, followed by serial dilutions. For dried probiotic films, 1 g of film was dissolved in 9 mL sterile Butterfield’s phosphate buffer, vortexed frequently for 30 min, then treated at 70 °C in the water bath for 30 min to stimulate spore activation prior to enumeration. The suspension was used as 10^−1^ dilution and appropriate serial dilution with Butterfield’s phosphate buffer. The dilution was cultured on MRS agar and incubated at 37 °C for 72 h. Counts of probiotics were performed in duplicate (*n* = 3) and expressed as log CFU/mL (film solution) or log CFU/g (films).

### 2.11. Survivability of Probiotic During Storage

The survivability of probiotics during storage was determined by dissolving 1 g of film in 9 mL of sterile Butterfield’s phosphate buffer solution. The mixture was vortexed frequently for 30 min, then placed in a water bath at 70 °C for 30 min and allowed to cool. This solution was used as the 10^−1^ dilution. Appropriate serial dilutions were prepared using Butterfield’s phosphate buffer solution, and each dilution was cultured on MRS agar and incubated at 37 °C for 72 h. Probiotic counts were performed in duplicate, with three replications, and expressed as log CFU/g. Observations were recorded on days 0, 15, and 30 (modified from [14]).

### 2.12. Statistical Analysis

Statistical analysis was carried out based on one-way analysis of variance (ANOVA) using IBM SPSS statistic 22. Tukey’s multiple test range (*p* < 0.05) was used to detect significant differences among values.

## 3. Results and Discussion

### 3.1. Fourier-Transform Infrared Spectroscopy (FTIR)

**Figure 1:**
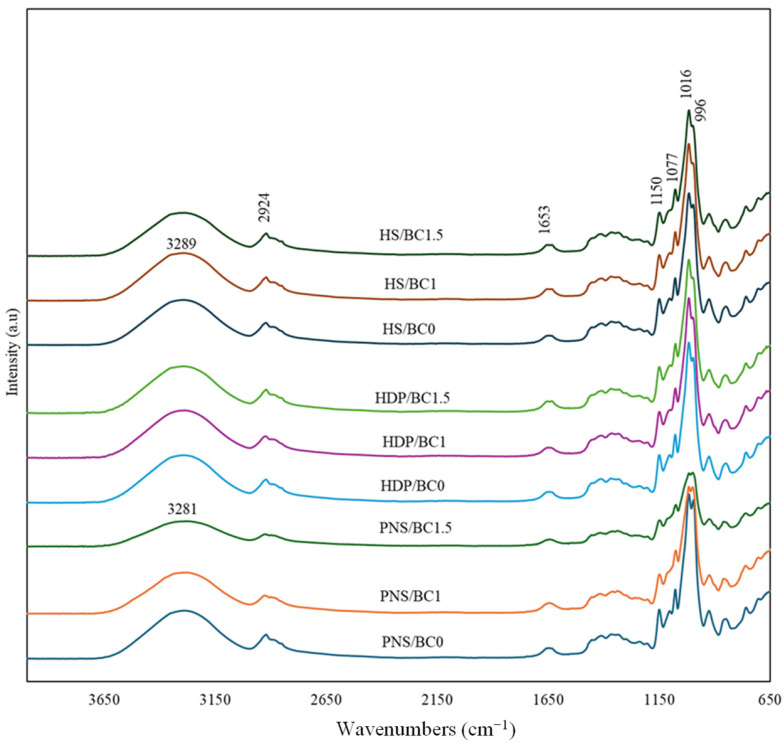
FTIR absorption spectra of edible films with different types of pregelatinized starch incorporated with *B. coagulans* at different concentrations (0, 1 and 1.5%).

The infrared absorption spectra of films containing different concentrations of BC powder are shown in Figure 1. All samples showed identical IR absorption peaks in the fingerprint region of polymers (500–1500 cm^−1^), indicating that BC incorporation did not significantly affect the chemical bonding of starch molecules. The range between 800–1500 cm^−1^ corresponds to the carbohydrate fingerprint region [22]. The most prominent peaks appeared at 1016 cm^−1^ and 996 cm^−1^, associated with the amorphous phase and water-sensitive regions, respectively [23]. Differences in pregelatinized starch types did not significantly affect these peaks. The intensity of the 1016 cm^−1^ peak increased from native to gelatinized starch probably due to the loss of ordered structure and decreased from gelatinized starch to retrograde starch due to reordering of the structure [24]. The addition of BC powder had less effect on the intensity of these bands on HDP and HS films but clearly decreased their intensity on PNS/BC1 and PNS/BC1.5 films, with the decrease becoming greater as higher concentration is added. The decrease in this FTIR peak suggests that interactions between BC powder and starch components modified the amorphous structure of the starch matrix. This is possibly due to lactose crystallization from the BC powder during the drying process. Lactose can crystallize as water is released, transforming from a high-moisture-content amorphous solid to a low-moisture-content crystalline form [25]. This transformation led to a reduction in amorphous regions and the formation of a more ordered structure. In the case of HS and HDP starches, the bulky hydroxypropyl groups hinder molecular reordering, leading to increase motional freedom of starch chains in the amorphous region [26,27]. The stability of this peak in HDP starch with or without BC powder, is possibly due to phosphate cross-linking, which reinforces inter- and intra-molecular bonding within the starch granules, thereby strengthening and stabilizing the granules. The presence of phosphate groups also restricts the mobility of cross-linked amylopectin, suppressing retrogradation in the starch matrix [28]. The incorporation of BC powder into PNS films, indicated by changes at 1016 cm^−1^ and 996 cm^−1^, affected the molecular reordering of starch.

The broad absorption bands between 3000 cm^−1^ and 3700 cm^−1^, corresponding to O-H stretching vibrations from starch and glycerol interactions, are associated with free, inter- and intra-molecular hydroxyl groups [3,29,30]. Although the different types of pregelatinized starch did not significantly affect this band, incorporating 1% and 1.5% BC into PNS films reduced the band intensity and shifted the peak from 3285 cm^−1^ to 3281 cm^−1^. In HDP films, adding 1% BC caused no peak shift, while 1.5% BC shifted the peak to a higher wavenumber (3288 cm^−1^ to 3294 cm^−1^). In HS films, BC addition slightly intensified the bands. A 1% addition shifted the peak upward (3288 cm^−1^ to 3289 cm^−1^), while 1.5% caused a slight downward shift (3288 cm^−1^ to 3287 cm^−1^). These changes may be attributed to hydroxyl groups in the lactose and maltodextrin coating on the probiotics powder. The observed decrease in absorbance, despite increased hydroxyl group content, may result from water binding to these groups, reducing the number of free O–H bonds [31]. While no new peaks were observed upon BC incorporation, the data suggest altered intermolecular interactions. The concentration of BC powder influenced the structural properties of both PNS and HS films through hydrogen bonding between starch matrix and probiotic components.

### 3.2. Microstructures

**Figure 2:**
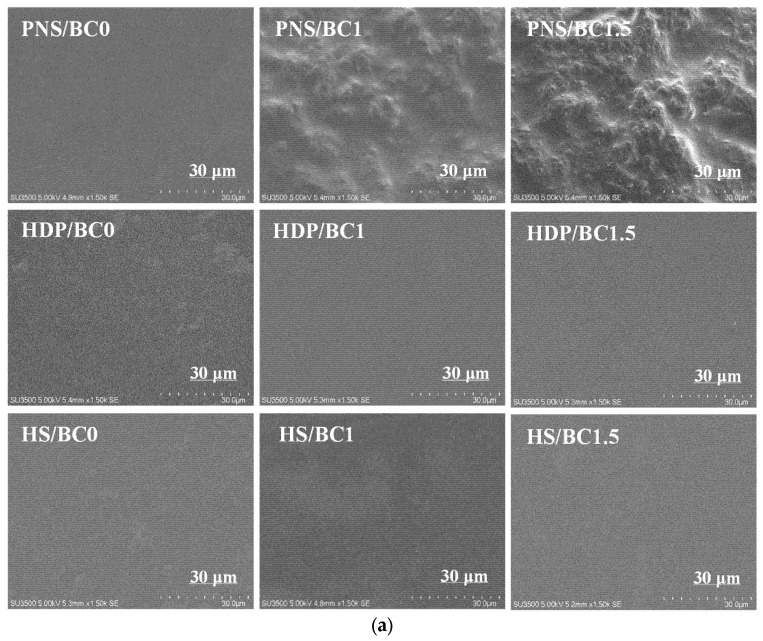

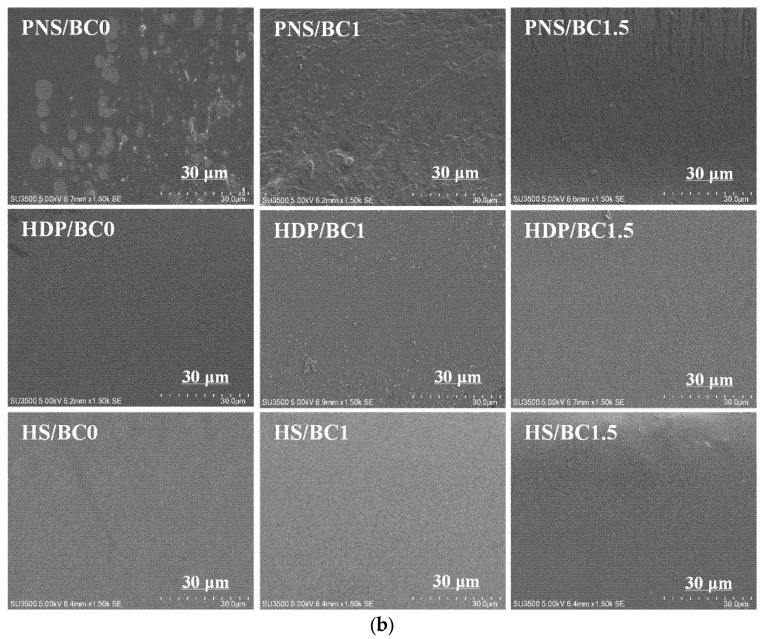
SEM microstructures on (**a**) surface and (**b**) cross-section of edible films with different types of pregelatinized starch incorporated with *B. coagulans* at different concentrations (0, 1 and 1.5%).

SEM was used to visualize the surface and cross-section microstructures of pregelatinized starch films incorporated with BC powder. The SEM images of the surface and cross-section of the films are presented in Figure 2a and Figure 2b, respectively. The PNS film exhibited a smooth surface, whereas the addition of BC powder (PNS/BC1 and PNS/BC1.5) resulted in a rougher surface with visible aggregates with increasing BC concentrations. These results align with the findings of Tan, et al., who reported that native cassava starch and carboxymethyl cellulose-based films containing *Bacillus* powder exhibited rougher surfaces and more uneven particles than control films [32]. This surface roughness suggests a degree of immiscibility between BC powder and pregelatinized native starch. This observation is supported by the FTIR results, where the addition of probiotic powder extended hydrogen bonding, weakening molecular interactions and promoting surface aggregation or clumping of starch granules. In contrast, HDP and HS films, both with and without BC exhibited smooth, homogeneous, and uniform surfaces. This suggests that the addition of BC powder did not disrupt the microstructure of HDP and HS films, indicating good dispersion and compatibility with the film matrix. Hydroxypropilation has been reported to destabilize the amorphous region of the starch granules and increase plasticization [28], which also contributes to mechanical properties, as discussed later. Both HDP and HS starches have undergone hydroxypropylation, a modification that introduces bulky hydroxypropyl groups. These groups create steric hindrance, which disrupts the alignment of starch chains and prevents aggregation [33].

The cross-section of PNS films, both with and without BC powder, exhibited heterogeneous structures probably due to the re-ordering of the starch structures. Pregelatinization treatment disrupts the granular structure of starch, leading to complete granular fragmentation. Starch leached from the granules during gelatinization plays a crucial role in the formation of the ordered structure [34,35]. HDP and HS films, both with and without BC, exhibited a relatively smooth surface morphology. The presence of hydroxypropyl groups in HDP and HS likely hindered the proper alignment of starch chains during aggregation and crystallization, as previously reported [36]. This homogeneous structure indicates high miscibility among all components within the HDP and HS films. However, the findings also reveal potential differences in the compatibility of BC powder with various starch-based film matrices. Due to their smooth appearance and structural integrity, HDP and HS matrices films incorporating BC powder show promise for use as edible coatings with desirable visual appeal.

### 3.3. Light Transmittance

**Figure 3:**
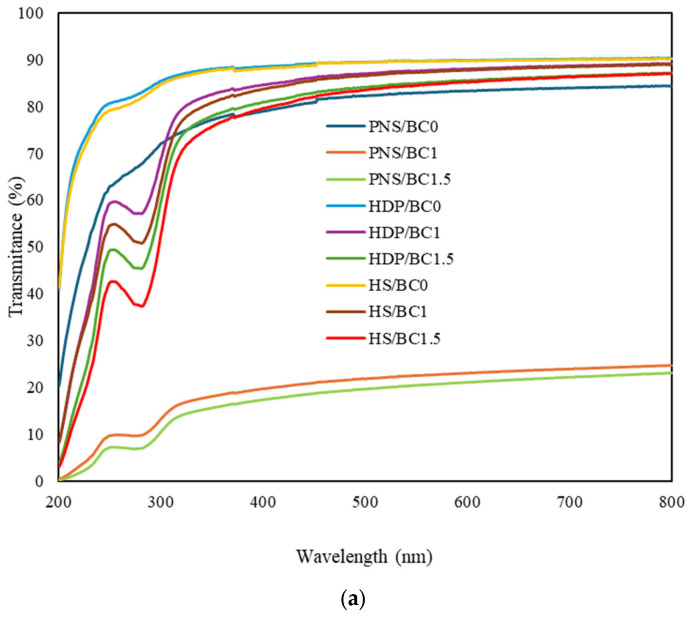

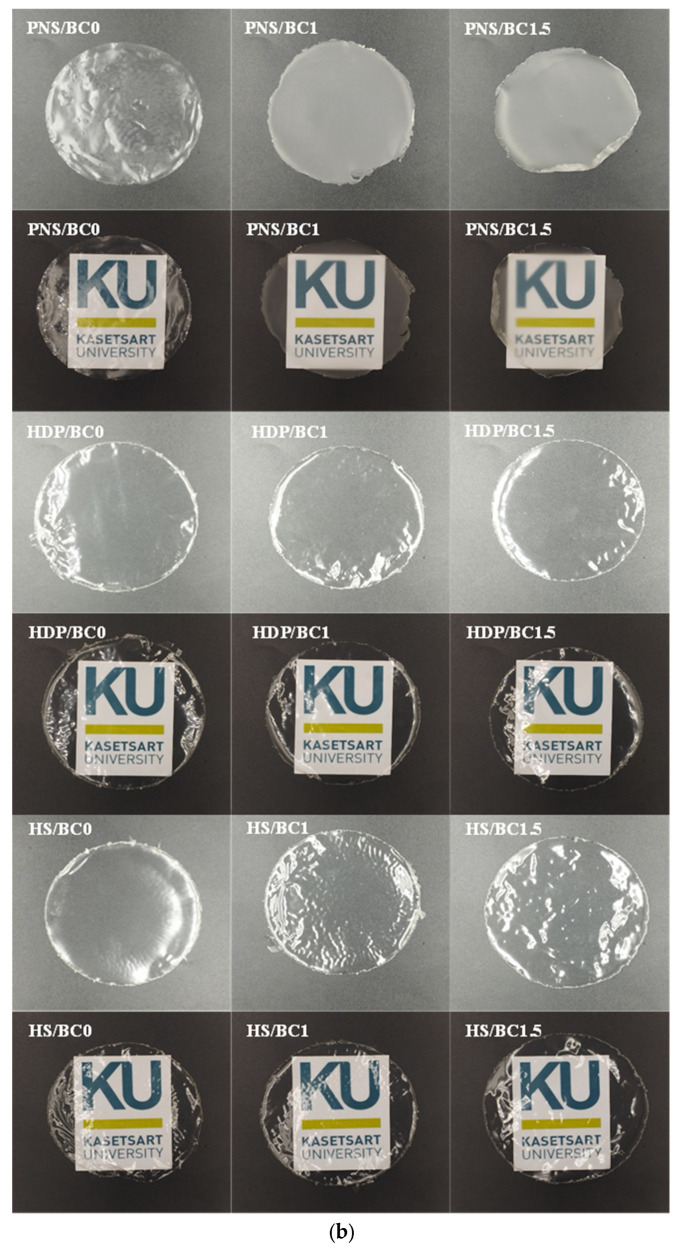
(**a**) Light transmittance and (**b**) appearance of edible films with different types of pregelatinized starch incorporated with *B. coagulans* at different concentrations (0, 1 and 1.5%).

The light transmittance and visual appearance of the films are presented in Figure 3a and Figure 3b respectively. The addition of BC powder significantly decreased the light transmittance of PNS film, resulting in a turbid appearance. Incorporating 1% BC reduced transmittance by up to 59.8%, while 1.5% led to a reduction of up to 61.3%. According to general classification standards for polymeric food packaging, these values fall below 80% and thus the PNS/BC films are more accurately categorized as translucent. The photographic contrast observed between the black backgrounds and card may contribute to a visual overestimation of clarity. This decrease transmittance is likely attributed to structural reordering between the PNS matrix and probiotic powder, as indicated by reduced IR spectral intensity and increased surface roughness observed in SEM images. These findings suggest that interactions between starch components and BC powder during the drying process lead to the formation of internal structures that scatter light [37]. In contrast, BC incorporation into HS and HDP films resulted in only slight reductions in light transmittance. Regardless of BC content, HDP and HS films maintained a high level of transparency. This may be explained by the presence of bulky hydroxypropyl groups in the modified starches, which create steric hindrance and prevent starch chain association, thereby reducing opacity [38]. Overall, the addition of probiotic powder significantly influenced the optical properties of PNS films, while HDP and HS matrices provided improved transparency. These results support the potential application of probiotic-loaded films as functional packaging materials with light barrier properties.

### 3.4. Mechanical Properties

**Figure 4:**
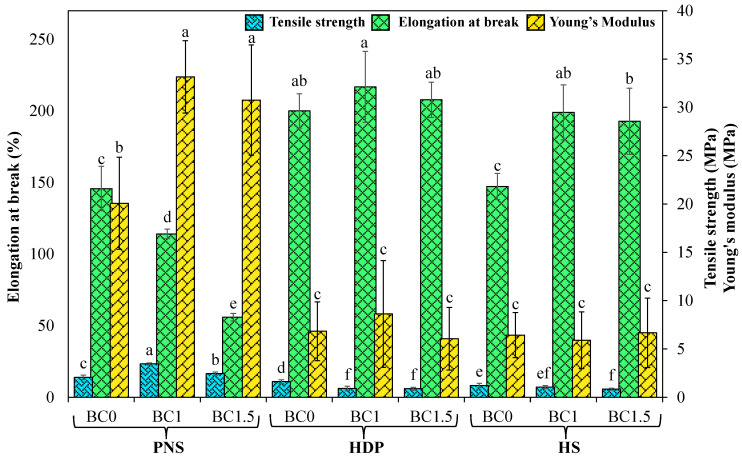
Mechanical properties of edible films with different types of pregelatinized starch incorporated with *B. coagulans* at different concentrations (0, 1 and 1.5%). Statistically significant differences were analyzed by one-way ANOVA with Tukey’s multiple test (*p* < 0.05). Mean ± SD values with different lowercase letters (^a, b, c, d, e, f^) within the same parameter (Tensile Strength, Elongation at Break, or Young’s Modulus) indicate significant differences between formulations (*p* < 0.05).

Figure 4 presents the tensile strength (TS), elongation at break (EB), and Young’s modulus (YM) of the films. The addition of BC powder significantly (*p* < 0.05) increased the TS and YM while decreasing the EB of the PNS films. SEM analysis revealed that the presence of probiotic powder up to 1.5% led to aggregation on the PNS film surface while enhancing compactness within the film matrix. This is supported by FTIR analysis, which showed a shift of the H-bond peak to lower wavenumbers, indicating stronger hydrogen bonding. These enhanced interactions between the polymer matrix and probiotic powders that restricted molecular mobility, contributing to increased mechanical strength but decreased flexibility of the film. Li, et al. reported that *Lactobacillus plantarum* was evenly distributed within the film matrix, resulting in a more compact and an increasing TS value [37]. Similarly, Shahrampour, et al. found that the addition of *L. plantarum* increased the TS due to hydrogen bonding between the bacteria and film molecules, which reduced intermolecular space [39]. In contrast, BC powder decreased the TS of HDP and HS films and had no significant effect on the YM, while significantly (*p* < 0.05) increasing the EB at a 1% concentration. Moreover, HS and HDP films exhibited higher EB values than PNS films, with or without BC powder. Higher EB values indicate increased flexibility, which may result from the plasticizing effect of bulky hydroxyl groups. Woggum, et al. similarly observed an increase in EB following hydroxypropylation of rice starch [40]. Hydroxypropyl groups interfere with amylose aggregation and inhibit the formation of junction zones [41]. These findings underscore the role of starch type in modulating the mechanical properties of *B. coagulans*-incorporated films.

### 3.5. Surface Hydrophobicity

**Figure 5:**
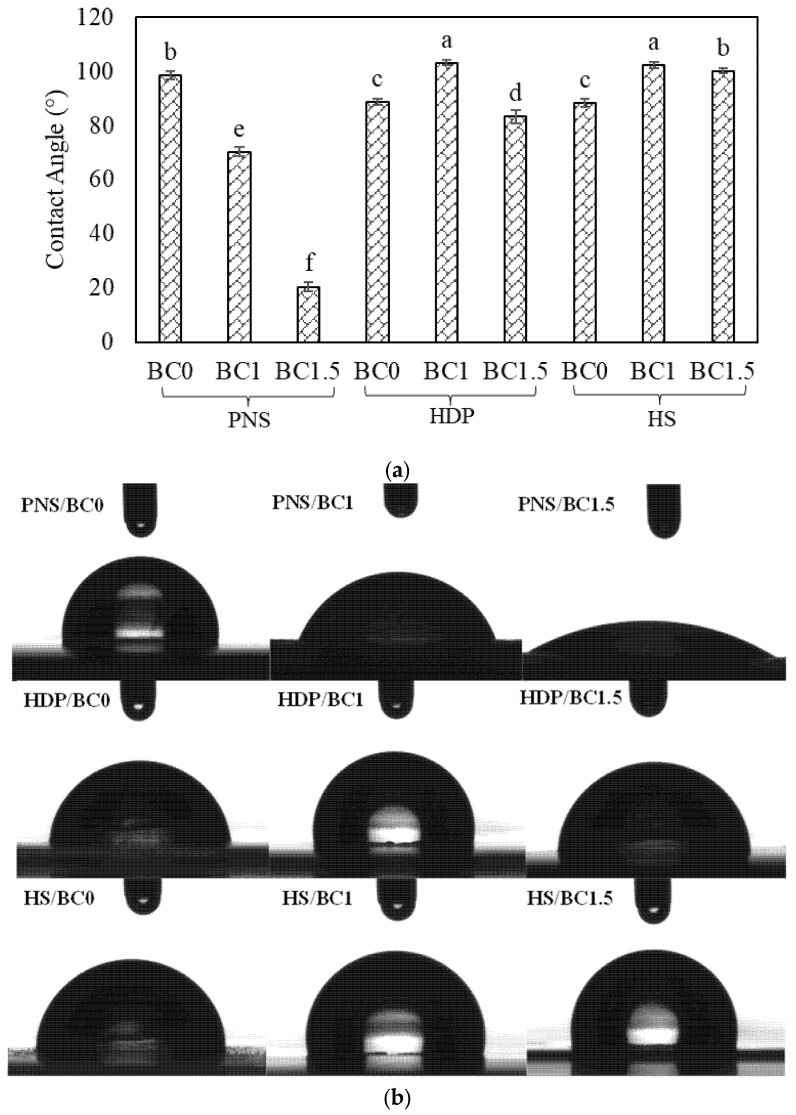
(**a**) Surface hydrophobicity and (**b**) image of water drop on edible films with different types of pregelatinized starch incorporated with *B. coagulans* at different concentrations (0, 1 and 1.5%). Statistically significant differences were analyzed by one-way ANOVA with Tukey’s multiple test (*p* < 0.05). Mean ± SD values with different lowercase letters (^a, b, c, d, e, f^) indicate significant differences between formulations (*p* < 0.05).

The contact angle (CA) and corresponding water drop images on the film are shown in Figure 5a and Figure 5b, respectively, illustrating the surface hydrophobicity of pregelatinized starch films with and without BC powder. The PNS film exhibited the highest CA, reflecting a more hydrophobic surface compared to HDP and HS films. This difference is likely attributed to the presence of bulky hydroxypropyl groups in HDP and HS, which facilitate water penetration into the starch granules [36]. The addition of BC powder significantly (*p* < 0.05) decreased the CA of the PNS film, with higher concentrations resulting in lower contact angles. This may be due to increased surface roughness, which promotes water migration. Chen, et al. similarly reported that zein films exhibited greater wettability than starch films due to their microporous surface and the presence of hydrophilic groups such as zein and glycerol, which tend to localize at the surface after the solvent evaporation and coagulation [42]. In this study, aggregates observed on the film surface in SEM images may contain hydrophilic groups that contributed to enhanced wettability. Interestingly, the addition of 1% BC powder significantly (*p* < 0.05) increased the CA of HDP films, whereas 1.5% addition reduced the CA. In HS films, BC powder addition significantly (*p* < 0.05) increased surface hydrophobicity. These results suggest that BC powder up to 1% may enhance hydrophobicity in HDP and HS films. Similarly, Tan, et al. observed contact angle values exceeding 90°, indicating increased hydrophobicity in films containing with *B. amyloliquefaciens* [32]. Overall, these findings highlight the complex interaction between starch type, BC concentration, and surface properties. While BC powder generally increased hydrophilicity in PNS films, it effects on HDP and HS films was more variable, suggesting the need for further investigation into the underlying mechanisms.

### 3.6. Water Solubility

**Figure 6:**
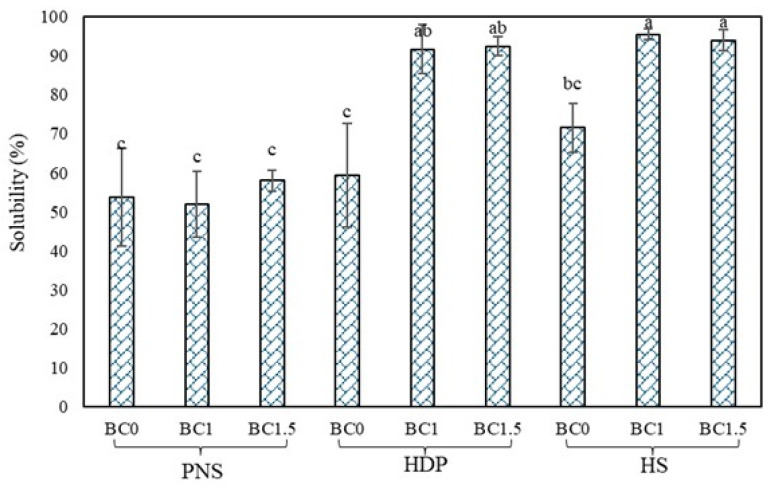
Water solubility of edible films with different types of pregelatinized starch incorporated with *B. coagulans* at different concentrations (0, 1 and 1.5%). Statistically significant differences were analyzed by one-way ANOVA with Tukey’s multiple test (*p* < 0.05). Mean ± SD values with different lowercase letters (^a, b, c^) indicate significant differences between formulations (*p* < 0.05).

The solubility of pregelatinized starch films with and without BC powder is presented in Figure 6. The solubility values of all control films (without BC powder) were not significantly different (*p* > 0.05). However, a slight increase in solubility was observed in HS films, probably due to the bulky hydroxypropyl groups facilitating easier water access to starch granules [36]. Although HDP also contains hydroxypropyl group, its cross-linked structure may restrict water mobility, resulting in lower solubility compared to HS [27]. Among all control films, solubility ranged from 53.65–71.56%. These findings confirm that pregelatinized starches exhibit higher solubility than native starch, due to the partial breakdown of amylose and amylopectin during the pre-gelatinization process. This disruption reduces crystallinity and improves water absorption capacity, contributing to good cold-water swelling [4]. Garcia, et al. similarly reported rapid disintegrating and good film integrity using gelatin and pregelatinized cassava starch blends [43]. Interestingly, although PNS films incorporated with BC powder showed high surface wettability (CA results), solubility did not increase accordingly. Contact angle measurements reflect initial surface wettability, while solubility involves bulk water penetration and matrix disintegration. This discrepancy may result from surface aggregates in PNS films that hinder water penetration, thus reducing water diffusion into the film matrix. IR analysis showed a reduction in free -OH groups in maltodextrin, which may weaken water affinity and slows down polymer chains release, resulting in lower solubility [44].

Conversely, BC addition significantly increased (*p* < 0.05) the solubility of HDP and HS films. The high solubility of HDP and HS films may be attributed to their internal hydrophilic functionality and modified starch structure. The I_996_ FTIR peak, known to be water-sensitive [23], was more pronounced in these films that indicating higher hydrophilicity. This is possibly due to the interaction between hydroxypropyl groups and BC components, which increased the availability of free –OH groups and water absorption. These findings suggest that both starch type and BC incorporation influence the solubility behavior of probiotic films. Overall, pregelatinized probiotic starch films show strong potential as packaging materials for applications involving water-soluble delivery.

An integrated evaluation of film structure and functionality highlights the influence of surface microstructure on film performance. SEM results showed that the addition of BC to PNS films induced surface roughness and visible aggregation. This correlated with a significant reduction in light transmittance due to increased light scattering. Although PNS films with BC showed high surface wettability, their solubility did not increase accordingly. This is possibly due to the aggregated surface limited uniform water interaction with the matrix. In contrast, HDP and HS films maintained smooth, homogeneous surfaces with BC addition, which contributed to higher transparency and enhanced solubility. These findings suggest that matrix compatibility and surface morphology are key factors influencing the functional behavior of probiotic-loaded starch films.

### 3.7. Water Vapor Permeability

**Figure 7:**
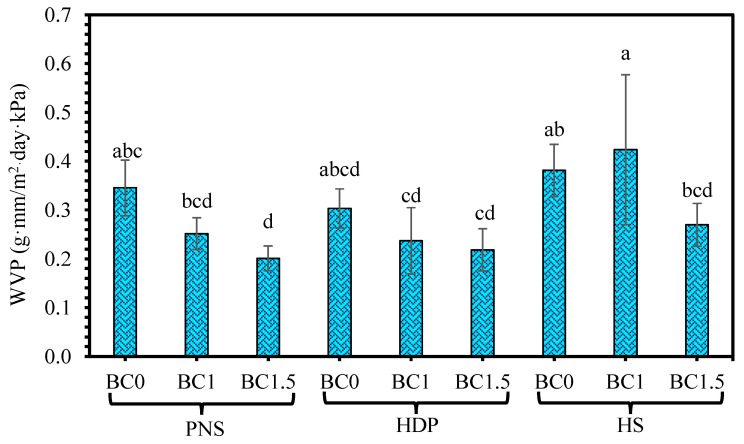
Water vapor permeability of edible films with different types of pregelatinized starch incorporated with *B. coagulans* at different concentrations (0, 1 and 1.5%). Statistically significant differences were analyzed by one-way ANOVA with Tukey’s multiple test (*p* < 0.05). Mean ± SD values with different lowercase letters (^a, b, c, d^) indicate significant differences between formulations (*p* < 0.05).

Water vapor permeability (WVP) involves sorption, diffusion, and adsorption and is primarily governed by interactions between the polymer matrix and water molecules. The WVP results are shown in Figure 7. No significant differences (*p* > 0.05) were observed in WVP among the control films or those containing various concentrations of BC. These results suggest that water vapor transmission was mainly influenced by diffusion through the film matrix. The abundance of hydroxyl groups in starch, which are highly hydrophilic, likely facilitates water vapor diffusion [45]. In contrast, Orozco-Parra, Mejía and Villa found that adding *Lactobacillus casei* significantly reduced WVP, possibly due to probiotic clustering disrupting the polymeric network and complicating water passage [9]. Similarly, Li, Ma, Ji, Sameen, Ahmed, et al. reported that probiotics acted as discontinuous particles in the film matrix, reducing polymer chain mobility and thus lowering WVP [37]. In the present study, the interaction between *B. coagulans* and the starch matrix may not have been sufficient to cause comparable structural disruption.

### 3.8. Viability of B. coagulans Before and After Drying Process

**Figure 8:**
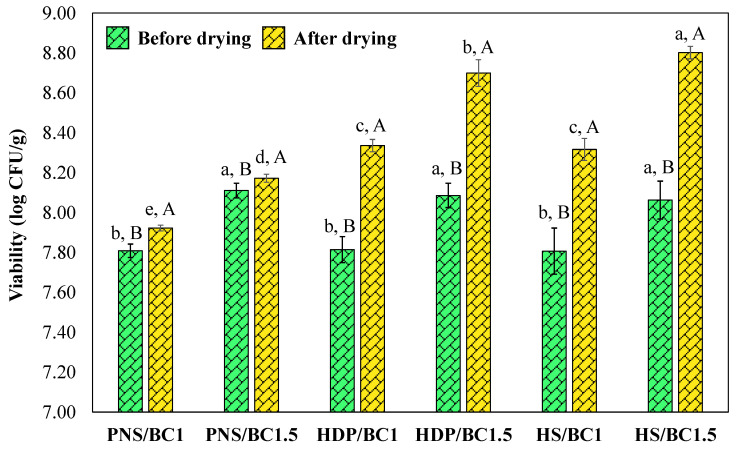
Viability of *B. coagulans* before and after the drying process. Data are expressed as mean ± standard deviation. Statistically significant differences were analyzed by one-way ANOVA with Tukey’s multiple test (*p* < 0.05). Mean ± SD values before and after drying process with different lowercase letters (^a, b, c, d, e^) indicate significant differences between formulations (*p* < 0.05). Different uppercase letters (^A, B^) within each formulation indicate significant differences between before and after the drying process (*p* < 0.05).

The viability of *B. coagulans* in both the film solution and the edible film is shown in Figure 8. The results indicate that *B. coagulans* viability was significantly influenced (*p* < 0.05) by both the probiotic concentration and the type of starch used. Higher concentrations of *B. coagulans* in the film solution led to higher viable counts in the edible films. Viable cell counts in the film solutions ranged from 7.81 ± 0.03 to 8.11 ± 0.04 log CFU/g. The drying process resulted in a significant (*p* < 0.05) increase in CFU/g counts of *B. coagulans*, consistent with previous findings on other *Bacillus* species [32]. This is attributed to a concentration effect due to water removal during drying. In addition, it could be due to the high resilience of *B. coagulans* in the production and storage process [46]. Although drying typically induces osmotic and oxidative stress that can impair bacterial viability [32], *B. coagulans* were able to survive within the film matrix. This suggests that pregelatinized cassava starches have the potential to protect probiotics. However, the lowest viable counts were observed in films incorporating pregelatinized native starch (PNS), likely due to its lower hydroxyl group content, as evidenced by IR spectroscopy, which may have reduced solubility and limited the release of probiotics from the films. This finding suggests that a higher hydroxyl groups density in the starch matrix may enhance probiotic protection [39].

### 3.9. Survivability of B. coagulans During Storage

The survivability of probiotics during storage is crucial to ensure their effectiveness in delivering live cells to the host and providing intended health benefits. As shown in Table 1, *B. coagulans* exhibited exceptional stability within the edible films over a 90-day storage period at 25 °C, maintaining viable counts between 7 and 8 log CFU/g. This high survivability aligns with previous studies demonstrating the resilience of *B. coagulans* in various delivery systems. For instance, Medeiros, et al. reported similar stability in alginate films [16], while Gholam-Zhiyan, et al. observed higher survivability for *B. coagulans* compared to *L. plantarum* in milk protein concentrate films [8]. This remarkable stability is attributed to the spore-forming nature of *B. coagulans*, which enables it to withstand harsh environmental conditions [47]. In addition, the starch matrix likely provides further protection, supporting the consistent delivery of viable cells to the target site. These findings suggest that pregelatinized cassava starches enhance the initial survival of *B. coagulans* after drying and help maintain its viability during storage. This study highlights their dual function in promoting both microbial viability and long-term stability within the film matrix.

## 4. Conclusions

This study successfully demonstrated the incorporation of BC into three distinct pregelatinized starch films. While the type of starch did not drastically alter the overall structure, it significantly influenced specific film properties. The addition of BC resulted in varied effects depending on the starch matrix. Notably, BC significantly enhanced the tensile strength of PNS films, roughened their surface and increased opacity, while HDP and HS films retained smoother surfaces and higher transparency. The solubility of PNS films was not affected by the addition of BC powder but increased in HDP and HS films. Importantly, *B. coagulans* exhibited excellent viability throughout the film processing and 90 days of storage, highlighting the potential of pregelatinized cassava starch films as effective probiotic carriers. These findings suggest promising applications in ready-to-eat food systems (such as edible wraps or snack coatings), functional pet feed products or beverage applications. Future research is recommended to explore the release kinetics of *B. coagulans* in water or gastrointestinal environments to confirm the functional efficacy of this system as a probiotic delivery carrier.

## Figures and Tables

**Table 1:** Survival of total probiotic cells (*Bacillus coagulans*) in film solution and the storage viability of probiotic cells entrapped in films during storage at room temperature and 50% RH.

Formulation	*Bacillus coagulans* in Film Solution (log CFU/g)	*Bacillus coagulans* in Edible Film (log CFU/g)			
		Day 0	Day 15	Day 30	Day 90
PNS/BC0	ND	ND	ND	ND	ND
PNS/BC1	7.81 ± 0.03 ^b^	7.92 ± 0.02 ^eA^	7.89 ± 0.09 ^dA^	8.00 ± 0.07 ^dA^	8.01 ± 0.04 ^dA^
PNS/BC1.5	8.11 ± 0.04 ^a^	8.17 ± 0.02 ^dA^	8.19 ± 0.03 ^cA^	8.22 ± 0.05 ^cA^	8.20 ± 0.07 ^cA^
HDP/BC0	ND	ND	ND	ND	ND
HDP/BC1	7.81 ± 0.07 ^b^	8.31 ± 0.03 ^cA^	8.37 ± 0.03 ^bA^	8.39 ± 0.02 ^bA^	8.27 ± 0.03 ^bcB^
HDP/BC1.5	8.09 ± 0.06 ^a^	8.70 ± 0.07 ^bA^	8.75 ± 0.04 ^aA^	8.76 ± 0.05 ^aA^	8.79 ± 0.06 ^aA^
HS/BC0	ND	ND	ND	ND	ND
HS/BC1	7.81 ± 0.12 ^b^	8.32 ± 0.06 ^cA^	8.36± 0.04 ^bA^	8.33 ± 0.02 ^bcA^	8.37 ± 0.04 ^bA^
HS/BC1.5	8.06 ± 0.10 ^a^	8.80 ± 0.03 ^aA^	8.80± 0.05 ^aA^	8.89 ± 0.08 ^aA^	8.83 ± 0.02 ^aA^

ND indicates no bacterial colony was detected on the agar plates. Mean ± SD values in the same column with different lowercase letters (^a, b, c, d, e^) indicate significant differences between formulations (*p* < 0.05). Mean ± SD values in the same row with different uppercase letters (^A, B^) indicate significant differences between storage days (*p* < 0.05). PNS: pregelatinized native starch, HDP: hydroxypropyl distarch phosphate, HS: hydroxypropyl starch, BC: *Bacillus coagulans*.

## Data Availability

The original contributions presented in the study are included in the article, further inquiries can be directed to the corresponding author.
